# Synthesis, Singlet Oxygen Photogeneration and DNA Photocleavage of Porphyrins with Nitrogen Heterocycle Tails

**DOI:** 10.3390/molecules16053488

**Published:** 2011-04-26

**Authors:** Yun-Man Zheng, Kai Wang, Tian Li, Xiu-Lan Zhang, Zao-Yin Li

**Affiliations:** 1 College of Chemistry and Molecular Sciences, Wuhan University, Wuhan 430072, China; E-Mail: yunman@126.com; 2 Key Laboratory for Green Chemical Process of Ministry of Education, Wuhan Institute of Technology, Wuhan 430074, China; E-Mail: maomaogo0128@126.com (T.L.); zhangxl@mail.wit.edu.cn (X.-L.Z.)

**Keywords:** porphyrin, singlet oxygen generation, DNA cleavage, heterocyclic thiol

## Abstract

Eight novel compounds were prepared by reaction of 5-(bromo- propoxyphenyl)-10,15,20-triphenylporphyrin with oxazole thiols, 1,3,4-oxadiazole thiols and 1,3,4-thiadiazole thiols, and their structures confirmed by UV-vis, IR, ^1^H-NMR, MS and elemental analysis. The assessment of indirectly measured ^1^O_2_ production rates against 5,10,15,20-tetraphenyl porphyrin (H_2_TPP) were described and the relative singlet oxygen production yields were: porphyrin **5** > porphyrins **1**, **3**, **4**, **6**-**8**, H_2_TPP > porphyrin **2**. Porphyrin **4** and porphyrin **7** showed substantial photocleavage activities toward DNA, with over 75% cleavage observed at 40 µM. It suggested that these those porphyrins with nitrogen heterocycle tails are potential photosensitive agents.

## 1. Introduction

The human vascular endothelial growth factor (VEGF) gene locates at chromosome 6P^21,3^, whose length is 28 Kb. The VEGF gene, of 14 Kb length, consists alternately of eight extrons and seven introns. The encoding product is a homogenous dimeric glycoprotein of 34–35 KD [1]. Against a target of VEGFR-2 tyrosine kinase VEGFR-2 plays a main receptor function in tumor angiogenesis mediated by VEGF, which prevents the activation of VEGFR-2 enzyme and limits VEGF signal transduction so as to inhibit tumor growth for cancer treatment. Compounds with oxazole, 1,3,4-oxadiazole and 1,3,4-thiadiazole structures display certain capability of acting in the VEGFR-2 tyrosine kinase domain and of binding VEGFR-2 in competition with ATP to prevent the growth of tumor vessels and cause tumor death [2,3,4]. So far, research on the use of angiogenesis inhibitors against the tumor metastasis has become a major topic.

Meanwhile, porphyrin-based compounds with unique structures have special affinity interactions with tumor cells and they can selectively remain in the tumor tissues [5,6]. Photodynamic therapy (PDT) is a promising therapy against cancer with intrinsic selectivity, which is used to eliminate deviant tissues, such as tumors. While gathering selectively around the tumors, the photosensitizers can absorb visible light under illumination and generate active oxygen (such as the singlet oxygen, ^1^O_2_, generated when porphyrins are photoactivated by absorption at the Soret band [7]), which leads to tumor cell death [8,9]. The ^1^O_2_ is produced by energy transfer from the photoexcited sensitizer (in its triplet state) to the ground-state (triplet) oxygen. This is known as the Type II photosensitization mechanism, which is considered to be more important in PDT [10,11]. The DNA photocleavage activity of a photosensitizer depends on its ^1^O_2_ yield [12,13].

Porphyrins linked with some anticancer drugs can effectively localize in tumor cells and increase the cell cytotoxicity through the synergistic effect of porphyrins and anticancer drugs [14]. To enhance targeted therapy role of pophyrins against the VEGFR-2 tyrosine kinase target some porphyrins with nitrogen heterocycle tails were prepared using as starting materials oxazole thiols, 1,3,4-oxadiazole thiols and 1,3,4-thiadiazole thiols linked by -O(CH_2_)_3_-S- spacer group (shown in Scheme 1). Their ^1^O_2_ productivity and DNA photocleavage activities were then investigated.

## 2. Results and Discussion 

### 2.1. Photogeneration of ^1^O_2_

DNA photocleavage of the porphyrins was the result of generating the singlet oxygen. 1,3-diphenylisobenzofuran (DPBF) was able to capture the ^1^O_2_ photogenerated by the porphyrins, which reduced its own light activity [15,16]. The relationship between A/A_0_ abosorbed by DPBF and illumination time indirectly reflected ^1^O_2_ yield of those porphyrins compared with H_2_TPP. 

As shown in Figure 1 for H_2_TPP and porphyrins **1**-**8**, the absorbance of DPBF at 418 nm decreased in the presence of each porphyrin with increasing illumination time. From the slope of the line, the relative rates of ^1^O_2_ photogeneration of the porphyrins could be compared. With the increasing of the line slopes, ^1^O_2_ yield was higher. It could be seen that the order was porphyrin **5** > porphyrins **1**, **3**, **4**, **6-8**, **H_2_TPP** > porphyrin **2**. The ability of photogenerating ^1^O_2_ by the photosensitizers might be greatly affected through the interaction between the chromophoric groups [17]. The yield of ^1^O_2_ photogeneration by porphyrins **1**, **3**, **4**, **6**-**8** was similar to that of H_2_TPP. The reason was that the conjugated system was not connected to the structure, but rather flexible chains were used, which made the porphyrins with oxazoles, oxadiazoles and thiadiazole rings show no significant change of ^1^O_2_ photogeneration compared to H_2_TPP. For copper (II) porphyrinate **2** no ^1^O_2_ photogenration was seen. This phenomenon has previously been observed in other paramagnetic metalloporphyrins with partially filled *d* orbitals, such as Ag^II^-TPP [18] and Co^II^-TCNPP [19], presumably due to a metal-facilitated relaxation of the porphyrin excited triplet state that precludes bimolecular quenching by oxygen. In contrast, Zn-porphyrin **5** showed a greater yield of ^1^O_2_, as other Zn (II)-porphyrin derivatives, and this is attributed to their correspondingly high triplet quantum yield, *Φ_T_* [20].

### 2.2. DNA photocleavage ability

The DNA photocleavage of porphyrins **1-8** and H_2_TPP was detected by monitoring the conversion of supercoiled form (form I) to the nicked circular form (form II). Only porphyrins **4**, **7** with cationic groups showed certain photocleavage activies towards DNA, with over 75% cleavage activities observed at 40 µM (see Figure 2). The reason might be that porphrins **4**, **7** with single cationic groups can bind the anionic area of DNA via electrostatic interactions. Although porphyrins **4**, **7** have relatively weaker electrostatic interaction with DNA compared with other cationic porphyrins, such as *meso*-tetrakis(*N*-methylpyridinium-4-yl)porpyrin, H_2_TMPyP, (98% cleavage activity at 2.5 µM [19]), porphyrins **4**, **7** also showed a good DNA photocleavage activity at higher concentrations. As for the remaining porphyrins and H_2_TPP, their insignificant DNA photocleavage activities (data not shown) were likely the result of a lack of a binding interaction with DNA and a more effectively oxidative attack from close range despite the high ^1^O_2_ yields. This suggests the importance of close-range interactions with the target for the effect of the drugs and the positive charge of porphyrin structures to affect the interaction with DNA [21,22].

In PDT, a good cellular uptake effect depends more on lipophilicity of a photosensitizer than on its ^1^O_2_ yield [23]. For example, H_2_TMPyP showed strong DNA binding and photocleavage activity, but its PDT efficacy was poor due to its poor cellular uptake. Hence, more porphyrins with both hydrophilic and lipophilic groups, such as porphyrins **4** and **7**, can be an effective PDT agent.

## 3. Experimental

### 3.1. Materials and methods

DMF was distilled from calcium hydride. 5-[4-(3-bromopropoxy)phenyl]-10,15,20-triphenyl- porphyrin and its Zn complex [24], 2-mercapto-5-phenyloxazole [3], 5-(3-pyridyl)-1,3,4-oxadiazole- 2-thione [25], 5-(3-pyridinyl)-1,3,4-thiadiazole-2- thione [26] and 5-(2-hydroxyphenyl)-2-thioxo- 1,3,4-oxadiazoline [27] were prepared according to the corresponding literature procedures. The other reagents were purchased from the China Sinopharm Company. NMR spectra were recorded with a Bruker ARX-300 (300 MHz) NMR spectrometer. Electronic absorption spectra in the UV/Vis region were recorded with a Shimadzu UV-PC 2401 spectrophotometer. The IR spectra (KBr pellets) were recorded with a Shimadzu FT-IR 3000 spectrometer. Melting points were measured with a Tianjing RY-1 melting point apparatus (the thermometer was not corrected). Elemental analyses were performed by using aVario ELIII elemental analyzer. High-resolution mass spectra (+ve mode, CDCl3) were recorded with a Bruker Autoflex MALDI-TOF mass spectrometer. All measurements were performed at ambient temperature (20 ± 2 °C) under atmospheric pressure.

### 3.2. Synthesis routes and procedures

#### 3.2.1. Preparation of porphyrin **1**

5-[4-(3-Bromopropoxy)phenyl]-10,15,20-triphenylporphyrin (106.5 mg, 0.14 mmol) and 2-mercapto-5-phenyloxazole (25 mg, 0.14 mmol) were dissolved in dry DMF (15 mL) and anhydrous K_2_CO_3_ (1 g) was added to the solution. Under nitrogen protection, the mixture was heated to 65 °C for 5 h. After cooling to room temperature, the reaction mixture was poured into water saturated with sodium chloride (30 mL) and filtered. The precipitate was purified on a silica gel column eluted with chloroform. The second fraction was the blue-violet title product. Yield: 90 mg, 76%. ^1^H-NMR (CDCl_3_) δ: −2.78 (2H, s, pyrrole, NH), 2.50~2.58 (2H, m, -CH_2_**CH_2_**CH_2_-), 3.57~3.60 (2H, t, *J* = 7.2 Hz, -CH_2_S-), 4.42 (2H, t, *J* = 7.2 Hz, -CH_2_O-), 7.35~7.38 (6H, m, oxazole-PhH and oxazole-H), 7.62~7.64 (3H, m, PorPhH_p_), 7.74~7.76 (8H, m, PorPhH_m_), 8.10~8.12 (2H, m, PorPhH_o_), 8.19~8.22 (6H, m, PorPhH_o_), 8.83 (8H, s, H_β_); UV-Vis (CHCl_3_, 20 °C), λ_max_ (log *ε*): 422 (5.58), 515 (4.18), 549 (3.85), 588 (3.70), 643 nm (3.34 dm^3^mol^−1^cm^−1^); IR (KBr), *ν*: 3316, 1600, 1242, 1173, 799 cm^−1^; HRMS: *m/z* = 847.3006 [M]^+^ (C_56_H_41_N_5_O_2_S: calcd. 847.2981, *Δ_m_* = 2.92 ppm) Anal. Calcd for C_56_H_41_N_5_O_2_S: C 79.31, H 4.87, N 8.26; found C 79.42, H 4.97, N 8.33. 

#### 3.2.2. Preparation of porphyrin **2**

The complex was prepared by heating porphyrin **1** (50 mg, 0.059 mmol) at reflux with an excess amount of copper (II) acetate in CHCl_3_/methanol for 3 h. It was then purified by column chromatography on silica gel with chloroform as eluent. Yield: 49 mg, 92%. UV-Vis (CHCl_3_, 20 °C), λ_max_ (log *ε*): 423 (5.86), 523 (4.83), 550 nm (4.52 dm^3^ mol^−1^ cm^−1^); IR (KBr), ν: 1601, 1508, 1242, 1175, 1000 (Cu^II^, OSMB), 812 cm^−1^; HRMS: *m/z* = 908.2150 [M]^+^ (C_56_H_39_N_5_O_2_SCu: calcd. 908.2121, *Δ_m_* = 3.24 ppm). Anal. Calcd for C_56_H_39_N_5_O_2_SCu: C 73.95, H 4.32, N 7.70; found C 73.84, H 4.39, N 7.61. 

#### 3.2.3. Preparation of porphyrin **3**

5-[4-(3-Bromopropoxy)phenyl]-10,15,20-triphenylporphyrin (106.5 mg, 0.14 mmol) and 5-(3-pyridyl)-1,3,4-oxadiazole-2-thione (27 mg, 0.14 mmol) were dissolved in dry DMF (15 mL) and anhydrous K_2_CO_3_ (1 g) was added to the solution. Under nitrogen protection, the mixture was heated to 65 °C for 5 h. After cooling to room temperature, the reaction mixture was poured into water saturated with sodium chloride (30 mL) and filtered. The precipitate was purified on a silica gel column eluted with chloroform. The second fraction was the blue-violet title product. Yield: 84 mg, 70%. ^1^H-NMR (CDCl_3_) δ: −2.78 (2H, s, pyrrole NH), 2.55~2.59 (2H, m, -CH_2_-), 3.71 (2H, t, *J* = 7.2 Hz, -CH_2_S-), 4.43 (2H, t, *J* = 7.2 Hz, -CH_2_O-), 7.43~7.47 (3H, m, PorPhH_p_), 7.74~7.76 (8H, m, PorPhH_m_), 8.10~8.13 (2H, m, -O-PorPhH_o_), 8.19~8.22 (6H, m, PorPhH_o_), 8.32~8.35 (1H, m, PyH_6_), 8.75~8.76 (2H, m, PyH_4-5_), 8.83 (8H, s, H_β_), 9.26 (1H, s, PyH_2_); UV-Vis (CHCl_3_, 20 °C), λ_max_ (log *ε*): 429 (5.66), 514 (5.19), 548 (4.76), 588 (4.23), 643 nm (3.68 dm^3^ mol^−1^ cm^−1^); IR (KBr) ν: 3313, 1600, 1242, 1177, 800 cm^−1^; HRMS: *m/z* = 849.2923 [M]^+^ (C_54_H_39_N_7_O_2_S: calcd. 849.2886, *Δ_m_* = 4.31 ppm). Anal. Calcd for C_54_H_39_N_7_O_2_S: C 76.30, H 4.62, N 11.53; found C 76.44, H 4.73, N 11.62.

#### 3.2.4. Preparation of porphyrin **4**

Porphyrin **3** (50 mg, 0.059 mmol) was added to CH_3_I (10 mL). After heating at reflux for 4 h under nitrogen, the solution was concentrated to dryness in vacuum. The precipitate was purified on a silica gel column and eluted with chloroform and methanol (v/v 20:1). The second fraction was the blue-violet title product. Yield: 53 mg, 90%. ^1^H-NMR (CDCl_3_) *δ*: −2.85 (2H, s, Pyrrole NH), 2.53~2.56 (2H, m, -CH_2_-), 3.73 (2H, t, *J* = 7.5 Hz, -CH_2_S-), 4.42 (2H, t, *J* = 7.5 Hz, -CH_2_O-), 4.46 (3H, s, -N-CH_3_), 7.39~7.43 (3H, m, PorPhH_p_), 7.72~7.75 (8H, m, PorPhH_m_), 8.11~8.12 (2H, m, -O-PorPhH_o_), 8.18~8.21 (6H, m, PorPhH_o_), 8.34~8.36 (1H, m, PyH_6_), 8.73~8.75 (2H, m, PyH_4-5_), 8.85 (8H, s, H_β_), 9.65 (1H, s, PyH_2_); UV-Vis (CHCl_3_, 20 °C), *λ_max_* (log *ε*): 426 (5.47), 514 (5.16), 548 (4.80), 586 (4.27), 645 nm (3.60 dm^3^mol^−1^cm^−1^); IR (KBr) *ν*: 3315, 1602, 1504, 1242, 1175, 805 cm^−1^; HRMS: *m/z* = 991.2181 [M]^+^ (C_55_H_42_IN_7_O_2_S: calcd. 991.2165, *Δ_m_* = 1.65 ppm). Anal. Calcd for C_55_H_42_IN_7_O_2_S: C 66.60, H 4.27, N 9.88; found C 66.54, H 4.33, N 9.91.

#### 3.2.5. Preparation of porphyrin **5**

Znic(II), 5-[4-(3-bromopropoxy)phenyl]-10,15,20-triphenylporphyrinate (50 mg, 0.061 mmol) and 5-phenyl-2-sulfydryl-1,3,4-oxadiazole (11 mg, 0.061 mmol) were dissolved in dry DMF (20 mL) and anhydrous potassium carbonate (1 g) was added to the solution. Under nitrogen protection, the mixture was heated to 65 °C for 4 h. After cooling to room temperature, the reaction mixture was poured into water saturated with sodium chloride (30 mL) and filtered. The precipitate was purified on a silica gel column and eluted with chloroform. The second fraction was the blue-violet title product. Yield: 40 mg, 71%. ^1^H-NMR (CDCl_3_), δ: 2.53~2.59 (2H, m, - CH_2_**CH_2_**CH_2_-), 3.68 (2H, t, *J* = 7.5 Hz, -CH_2_S-), 4.43 (2H, t, *J* = 5.1 Hz, -CH_2_O-), 7.28~7.29 (3H, m, oxadiazole-PhH_m, p_) 7.51~7.52 (3H, m, PorPhH_p_), 7.73~7.76 (8H, m, PorPhH_m_), 8.03~8.06 (2H, m, oxadiazole-PhH_o_), 8.10~8.13 (2H, m, -O-PorPhH_o_), 8.20~8.22 (6H, m, PorPhH_o_), 8.92 (8H, s, H_β_); UV-Vis (CHCl_3_, 20 °C), *λ_max_* (log *ε*): 427 (5.80), 521 (4.45), 554 nm (3.76 dm^3^ mol^−1^ cm^−1^); IR (KBr) ν: 3316, 1598, 1240, 1174, 996 (Zn^II^, OSMB), 810 cm^−1^; HRMS: *m/z* = 910.2103 [M]^+^ (C_55_H_38_N_6_O_2_SZn: calcd. 910.2068, *Δ_m_* = 3.87 ppm). Anal. Calcd for C_55_H_38_N_6_O_2_SZn: C 72.40, H 4.20, N 9.21; found C 72.53, H 4.31, N 9.25.

#### 3.2.6. Preparation of porphyrin **6**

5-[4-(3-Bromopropoxy)phenyl]-10,15,20-triphenylporphyrin (106 mg, 0.14 mmol) and 5-(3-pyridinyl)-1,3,4-thiadiazole-2-thione (27 mg, 0.14 mmol) were dissolved in dry DMF (15 mL) and anhydrous potassium carbonate (1 g) was added to the solution. Under nitrogen protection, the mixture was heated to 65 °C for 6 h. After cooling to room temperature, the reaction mixture was poured into water saturated with sodium chloride (30 mL) and filtered. The precipitate was purified on a silica gel column and eluted with chloroform. The second fraction was the blue-violet title product. Yield: 92 mg, 76%. ^1^H-NMR (CDCl_3_) δ: −2.78 (2H, s, pyrrole NH), 2.55~2.59 (2H, m, -CH_2_-), 3.76~3.81 (2H, t, *J* = 6.0 Hz, -CH_2_S-), 4.42~4.45 (2H, t, *J* = 6.0 Hz, -CH_2_O-), 7.28~7.31 (5H, m, PorPhH_p_ and PyH_4, 5_), 7.35~7.40 (1H, m, PyH_6_), 7.75~7.77 (8H, m, PorPhH_m_), 8.11~8.14 (2H, m, -O-PorPhH_o_), 8.20~8.23 (6H, m, PorPhH_o_), 8.53 (1H, s, PyH_2_), 8.84 (8H, s, H_β_); UV-Vis (CHCl_3_, 20 °C), *λ_max_* (log *ε*): 424 (5.66), 513 (4.32), 550 (3.85), 586 (3.77), 633 nm (3.56 dm^3^ mol^−1^ cm^−1^); IR (KBr) ν: 3315, 1601, 1503, 1243, 1176, 803 cm^−1^; HRMS: *m/z* = 865.2693 [M]^+^ (C_54_H_39_N_7_OS_2_: calcd. 865.2658, *Δ_m_* = 4.05 ppm). Anal. Calcd for C_54_H_39_N_7_OS_2_: C 74.89, H 4.54, N 11.32; found C 75.01, H 4.65, N 11.38.

#### 3.2.7. Preparation of porphyrin **7**

Porphyrin **6** (50 mg, 0.058 mmol) was added to CH_3_I (10 mL). After heating at reflux for 4 h under nitrogen, the solution was concentrated to dryness in vacuum. The precipitate was purified on a silica gel column and eluted with chloroform and methanol (v/v 20:1). The second fraction was the blue-violet title product. Yield: 54 mg, 92%. ^1^H-NMR (CDCl_3_) δ: −2.82 (2H, s, pyrrole NH), 2.50~2.55 (2H, m, -CH_2_-), 3.77 (2H, t, *J* = 7.5 Hz, -CH_2_S-), 4.40 (2H, t, *J* = 7.5 Hz, -CH_2_O-), 4.48 (3H, s, -N-CH_3_), 7.74~7.76 (11H, m, PorPhH_m_ and PorPhH_p_), 8.10~8.12 (2H, m, -O-PorPhH_o_), 8.19~8.22 (6H, m, PorPhH_o_), 8.83~8.85 (11H, m, H_β_ and PyH_4-6_), 9.70 (1H, s, PyH_2_); UV-Vis (CHCl_3_, 20 °C), *λ_max_* (log *ε*): 423 (5.70), 514 (4.40), 548 (3.80), 588 (3.69), 638 nm (3.40 dm^3^ mol^−1^ cm^−1^); IR (KBr) *ν*: 3313, 1600, 1242, 1177, 805 cm^−1^; HRMS: *m/z* = 1007.1962 [M]^+^ (C_55_H_42_IN_7_OS_2_: calcd. 1007.1937, *Δ_m_* = 2.53 ppm). Anal. Calcd for C_55_H_42_IN_7_OS_2_: C 65.53, H 4.20, N 9.73; found C 65.45, H 4.29, N 9.69.

#### 3.2.8. Preparation of porphyrin **8**

5-[4-(3-Bromopropoxy)phenyl]-10,15,20-triphenylporphyrin (106.5 mg, 0.14 mmol) and 5-(2-hydroxyphenyl)-2-thioxo-1,3,4-oxadiazoline (13 mg, 0.14 mmol) were dissolved in dry DMF (25 mL) and anhydrous potassium carbonate (1 g) was added to the solution. Under nitrogen protection, the mixture was heated to 65 °C for 5 h. After cooling to room temperature, the reaction mixture was poured into water saturated with sodium chloride (30 mL) and filtered. The precipitate was purified on a silica gel column and eluted with chloroform. The second fraction was the blue-violet title product. Yield: 130 mg, 61%. ^1^H-NMR (CDCl_3_) δ: −2.77 (4H, s, pyrrole NH), 2.44~2.48 (2H, m, -OCH_2_-**CH_2_**-CH_2_-S-), 2.54~2.57 (2H, m, -OCH_2_-**CH_2_**-CH_2_-O-), 3.63 (2H, t, *J* = 6.6 Hz, -OCH_2_-CH_2_-**CH_2_**-S-), 4.18 (2H, t, *J* = 5.1 Hz, -O**CH_2_**CH_2_CH_2_-O-Por), 4.49 (2H, t, *J* = 6.0 Hz, Por-O-**CH_2_**CH_2_CH_2_S-), 4.59 (2H, t, *J* = 6.0 Hz, Por-O-**CH_2_**CH_2_ CH_2_O-), 7.33~7.35 (4H, m, PhH_3-6_), 7.54~7.61 (6H, m, PorPhH_p_), 7.65~7.76 (16H, m, PorPhH_m_), 7.98~8.00 (4H, m, -O-PorPhH_o_), 8.05~8.07 (4H, m, -O-PorPhH_o_), 8.14~8.22 (12H, m, PorPhH_o_), 8.75~8.89 (16H, m, H_β_); UV-Vis (CHCl_3_, 20 °C), *λ_max_* (log *ε*): 421 (5.50), 520 (4.18), 552 (3.90), 589 (3.60), 651 nm (3.59 dm^3^mol^−1^cm^−1^); IR (KBr) ν: 3312, 1607, 1240, 1173, 798 cm^−1^; HRMS: *m/z* = 1535.5677 [M]^+^ (C_102_H_74_N_10_O_4_S: calcd. 1535.5649, *Δ_m_* = 1.83 ppm). Anal. Calcd for C_102_H_74_N_10_O_4_S: C 79.77, H 4.86, N 9.12; found C 79.68, H 4.91, N 9.20.

### 3.3. Measurement of singlet oxygen production rate

1,3-Diphenylisobenzofuran (DPBF) was used as a selective singlet oxygen (^1^O_2_) acceptor, which was bleached upon reaction with ^1^O_2_. Eight sample solutions of DPBF in DMSO (50 μM) containing, respectively no porphyrin (control sample), H_2_TPP (1 μM) and porphyrins **1**-**8** (1 μM) were prepared in the dark. Each sample container was covered with aluminum foil with a yellow filter (with cutoff wavelength <500 nm) on one side. The samples were then exposed to light (50 watt) through the filter. After irradiation, visible spectra of the sample solutions were measured spectrophotometrically. The normalized absorbances of DPBF at 418 nm in these samples were reported as a function of the photo-irradiation time. From this plot, the rates of ^1^O_2_ production of porphyrins **1**-**8** relative to those of H_2_TPP were determined.

### 3.4. DNA photocleavage assay

The DNA photocleavage activities of porphyrins **1**-**8** and H_2_TPP were measured using the plasmid DNA relaxation assay. Briefly, the plasmid DNA (pBluescript, 0.5 μg), enriched with the covalently-closed circular or supercoiled conformer (Form I), and the one-phor-all plus buffer (10 mM Tris-acetate, 10 mM magnesium acetate, 50 mM potassium acetate, pH 7.5) was vortexed. Aliquots of the DNA were pipetted into different Eppendorf tubes. Various amounts of autoclaved water (control sample) or prophyrins (test sample) were added into the Eppendorf tubes to give a final volume of 20 μL in each sample tube. The sample mixtures were then photo-irradiated at 400–450 nm for 60 min using a transilluminator (Vilber Lourmat) containing 4 × 15 W light tubes (Aqua Lux) with maximum emission at 435 nm. After photo-irradiation, 2 μL of the 6x sample dye solution (which contained 20% glycerol, 0.25% bromophenol blue and 0.25% xylene cyanol FF) was added to each Eppendorf tube and mixed well by centrifugation. The sample mixtures were loaded onto a 0.8% (v/v) agarose gel (Gel dimension: 13 cm × 10 cm), with 1× TBE buffer (89 mM Tris-borate, 1 mM EDTA, pH 8) used as supporting electrolyte, and electrophoresized at 1.3 Vcm^−1^ for 3 h using a mini gel set (CBS Scientific Co., Model No. MGU-502T). After electrophoresis, the gel was stained with 0.5 μg/mL ethidium bromide solution for 30 min and then destained using deionized water for 10 min. The resulting gel image was viewed under 365 nm and captured digitally using a gel documentation system (BioRad).

## 4. Conclusions

Eight porphyrins with nitrogen heterocycle tails were synthesized and characterized. Compared with H_2_TPP, the ^1^O_2_ yields of porphyrins **1**-**8** were measured indirectly and their DNA photocleavage activities were tested. Two porphyrins with cationic groups, **4** and **7**, which showed certain singlet oxygen yields and DNA photocleavage activities could be potenial photosensitizers. The *in vitro* PDT activities against VEGFR-2 receptors and tumor angiogenesis are currently under investigation.

## Figures and Tables

**Scheme 1:**
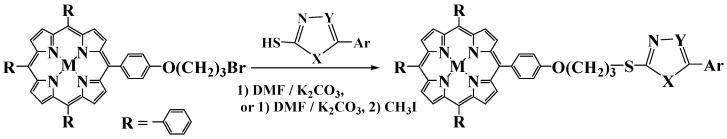
Synthesis of eight porphyrins with nitrogen heterocycle tails.

**Table:** 

Porphyrin	M	X	Y	Ar
**1**	2H	O	CH	-Ph
**2**	Cu	O	CH	-Ph
**3**	2H	O	N	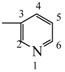
**4**	2H	O	N	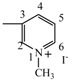
**5**	Zn	O	N	-Ph
**6**	2H	S	N	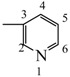
**7**	2H	S	N	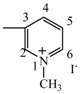
**8**	2H	O	N	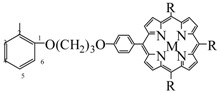

**Figure 1:**
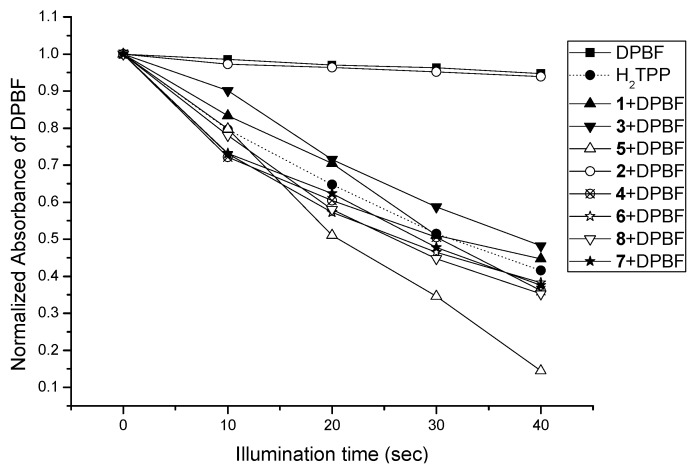
The relation between illumination time and DPBF’s absorption value ratio (*A/A_0_*).

**Figure 2:**
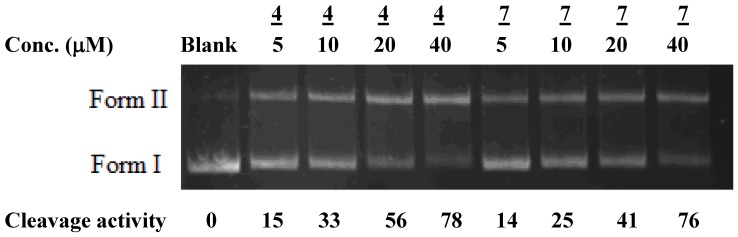
DNA photocleavage activity of porphyrin **4** (lanes 2-5) and porphyrin **7** (lanes 6-9) as a function of its concentration. Lane 1: DNA (Form I) control; lane 2, 6: 5 μM; lane 3, 7: 10 μM; lane 4, 8: 20 μM; lane 5, 9: 40 μM for porphyrin **4** and **7**. Photo-irradiation conditions: λ_irrad_ = 455 nm; duration, 60 min.
